# Genome-wide association studies for diabetic macular edema and proliferative diabetic retinopathy

**DOI:** 10.1186/s12881-018-0587-8

**Published:** 2018-05-08

**Authors:** Patricia S. Graham, Georgia Kaidonis, Sotoodeh Abhary, Mark C. Gillies, Mark Daniell, Rohan W. Essex, John H. Chang, Stewart R. Lake, Bishwanath Pal, Alicia J. Jenkins, Alex W. Hewitt, Ecosse L. Lamoureux, Philip G. Hykin, Nikolai Petrovsky, Matthew A. Brown, Jamie E. Craig, Kathryn P. Burdon

**Affiliations:** 1 Menzies Institute for Medical Research, University of Tasmania, Hobart, Tasmania Australia; 2 Department of Ophthalmology, Flinders University, Flinders Medical Centre, Adelaide, South Australia Australia; 3 Save Sight Institute, Clinical Ophthalmology and Eye Health, University of Sydney, Sydney, New South Wales Australia; 4 Department of Ophthalmology, Royal Melbourne Hospital, Parkville, Victoria Australia; 5 Academic Unit of Ophthalmology, Australian National University, Canberra, Australia; 6 School of Medical Sciences, University of NSW, Sydney, New South Wales Australia; 7 Medical Retina Service, Moorfields Eye Hospital, London, UK; 8 NHMRC Clinical Trials Centre, University of Sydney, Camperdown, New South Wales Australia; 9 St Vincent’s Hospital, Fitzroy, Victoria Australia; 10 Centre for Eye Research Australia, University of Melbourne, East Melbourne, Victoria Australia; 11 Singapore Eye Research Institute, Singapore, Singapore; 12 Department of Endocrinology, Flinders University, Flinders Medical Centre, Adelaide, South Australia Australia; 13 Institute of Health and Biomedical Innovation, Queensland University of Technology, Brisbane, Australia

**Keywords:** Genome-wide association study, Diabetic retinopathy, Macular edema, Genetics, Diabetes complications

## Abstract

**Background:**

Diabetic macular edema (DME) and proliferative diabetic retinopathy (PDR) are sight-threatening complications of diabetes mellitus and leading causes of adult-onset blindness worldwide. Genetic risk factors for diabetic retinopathy (DR) have been described previously, but have been difficult to replicate between studies, which have often used composite phenotypes and been conducted in different populations. This study aims to identify genetic risk factors for DME and PDR as separate complications in Australians of European descent with type 2 diabetes.

**Methods:**

Caucasian Australians with type 2 diabetes were evaluated in a genome-wide association study (GWAS) to compare 270 DME cases and 176 PDR cases with 435 non-retinopathy controls. All participants were genotyped by SNP array and after data cleaning, cases were compared to controls using logistic regression adjusting for relevant covariates.

**Results:**

The top ranked SNP for DME was rs1990145 (*p* = 4.10 × 10^− 6^, OR = 2.02 95%CI [1.50, 2.72]) on chromosome 2. The top-ranked SNP for PDR was rs918519 (*p* = 3.87 × 10^− 6^, OR = 0.35 95%CI [0.22, 0.54]) on chromosome 5. A trend towards association was also detected at two SNPs reported in the only other reported GWAS of DR in Caucasians; rs12267418 near *MALRD1* (*p* = 0.008) in the DME cohort and rs16999051 in the diabetes gene *PCSK2* (*p* = 0.007) in the PDR cohort.

**Conclusion:**

This study has identified loci of interest for DME and PDR, two common ocular complications of diabetes. These findings require replication in other Caucasian cohorts with type 2 diabetes and larger cohorts will be required to identify genetic loci with statistical confidence. There is considerable overlap in the patient cohorts with each retinopathy subtype, complicating the search for genes that contribute to PDR and DME biology.

**Electronic supplementary material:**

The online version of this article (10.1186/s12881-018-0587-8) contains supplementary material, which is available to authorized users.

## Background

Diabetic retinopathy (DR) is a common and potentially blinding complication of both type 1 and type 2 diabetes mellitus. The disease affects multiple vascular and neural cell types of the retina and is the leading cause of new cases of blindness in working aged adults [1]. The early non-proliferative DR (NPDR) stages are characterized by retinal microaneurysms, lipid and protein deposits and cotton wool spots due to damage to retinal vasculature. The later stage, characterised by neovascularisation of the retina is called proliferative DR (PDR). Patients may also develop diabetic macular edema (DME) characterized by build-up of fluid in and beneath the macula, affecting detailed central vision, with or without PDR or NPDR. The prevalence of any retinopathy among patients with diabetes is 40.3%, with a prevalence of 8.2% for sight threatening DR, classified as severe NPDR, PDR or DME [2].

Longer duration of diabetes, poor glycemic control, hypertension, dyslipidemia, central obesity, smoking and high blood pressure are well-documented risk factors for ocular diabetic complications [3, 4]. Genetic factors, however, also play an important role. Family studies have shown that siblings have a greater chance of developing DR, irrespective of other risk factors and the heritable component of DR has been calculated in the range of 25–50% [5, 6]. Candidate gene studies have identified numerous genetic variants that may contribute to risk, but have often been inconsistent [7, 8]. Genome-wide association studies (GWAS) are an efficient method to search for genetic variants associated with complex diseases, including DR.

Six GWAS for DR related phenotypes have been reported to date. The first was conducted in Mexican-Americans with type 2 diabetes with severe NDPR or PDR [9]. Subsequent studies in Taiwanese [10] and Japanese [11] participants used a similar disease definition whereas a study in Chinese patients was limited to PDR [12]. The most recent study in type 2 diabetes used a composite definition of blinding DR which included patients with severe NPDR, PDR or DME [13] and was conducted in Caucasian participants for the first time. Only one GWAS for DR has been reported in patients with type 1 diabetes [14]. This study was also conducted in a Caucasian cohort and included patients with DME and PDR as cases. Although many loci have been suggested through these studies, there has been limited success in replicating findings between studies, partly due to small study sizes increasing the likelihood of false positive findings and partly due to different ethnic groups and disease definitions under investigation.

Previous GWAS in Caucasian cohorts have, to date, only considered composite phenotypes. This may limit the results by creating heterogeneous groups with different underlying genetic risk factors. Therefore, we undertook a re-analysis of our previously reported GWAS in Australians with type 2 diabetes [13] stratified by patients with PDR and those with DME, separately. We then compared our findings to those reported in the only other published DR GWAS of Caucasian patients, reported by Grassi et al. [14].

## Methods

### Recruitment of participants and clinical data collection

In South Australia, ethics approval was obtained from the Southern Adelaide Health Service Flinders University Clinical Research Ethics Committee (Flinders Medical Centre and Repatriation General Hospital), and the Human Research Ethics Committees (HREC) of the Royal Adelaide Hospital and Queen Elizabeth Hospital. In New South Wales and Victoria, the HREC of Southeastern Sydney and Illawara Northern Hospital Network (Sydney Eye Hospital), and the HREC of Melbourne Health (Royal Melbourne Hospital) approved this study. The ACT Health HREC approved this study in the Australian Capital Territory. This retrospective, cross sectional study adhered to the tenets of the Declaration of Helsinki. Written informed consent was obtained from all study participants, who have been previously described [13, 15]. Briefly, patients with medically treated type 2 diabetes for at least 5-years were recruited and underwent a thorough ophthalmological examination. DR and DME was graded on clinical examination according to recognised severity scales based on ETDRS criteria [16]. Participants completed a detailed questionnaire and concurrent clinical measurements for blood pressure, renal function and HbA1c were obtained. Patients were classified as controls if they had no sign of DR or DME. Cases were defined as those with PDR and those with DME present in their worst affected eye, to be analyzed as two separate case cohorts. Patients with mild, moderate or severe NPDR were excluded from the current analysis although some patients with DME had NPDR concurrently. Those with both DME and PDR were included in both case cohorts.

### SNP genotyping and data analysis

Genotyping of single nucleotide polymorphisms (SNPs) across the whole genome have been previously described [13]. Briefly, genomic DNA extracted from whole blood from each individual was genotyped on the OmniExpress SNP array (Illumina). Standard quality control filters were applied to the genotype data and 617,130 SNPs were included in the subsequent analyses. Principal components analysis was conducted using Eigenstrat [17] and individuals falling greater than six standard deviations from the mean of each vector were removed. Q-Q and Manhattan plots were generated using the qqman package [18] in R (https://www.r-project.org/).

Demographic and clinical characteristics were compared using t-tests for continuous traits and chi-squared tests for dichotomous traits. Association of each SNP passing quality control with each DR phenotype (DME and PDR) was assessed using logistic regression adjusted for the covariates age, diabetes duration, sex, hypertension, nephropathy, HbA1c and the first three principal components, using PLINK [19]. *P*-values < 5 × 10^− 8^ were deemed to be significant at a genome-wide level. SNPs for comparison with other Caucasian GWAS were chosen from Grassi et al., 2011 [14]. For reported SNPs that were not directly genotyped in the current study, a proxy SNP with r^2^ > 0.8 with the reported SNP in HapMap was chosen as described previously [13]. Where no such SNP was available, the SNP with the smallest *p*-value within 50 kb from the reported SNP was selected. When assessing the eight previously reported loci a p-value < 0.0063 was considered significant under a Bonferroni correction for eight independent tests.

## Results

The DR phenotypes of all participants are shown in Table 1. The PDR cohort consisted of 176 cases (with or without DME) while the DME cohort had 270 cases (with or without PDR). 93 participants had both DME and PDR and were common to both case cohorts. The controls with no PDR or DME were also common to both groups.

**Table 1:** Retinopathy grading of study cohorts. All participants had type 2 diabetes

Retinopathy grading	N
No retinopathy (controls)	435
DME only	177
PDR only	83
DME and PDR	93

*DME* diabetic macular edema, *PDR* proliferative diabetic retinopathy, *N* number of participants

The demographics and clinical characteristics of both cases compared to controls are shown in Table 2. Well known DR risk factors including; duration of diabetes, hypertension, nephropathy and HbA1c were significantly different between the control and case groups in both cohorts. Age and sex were associated with PDR with cases being younger and more likely to be male than controls, but this association was not seen with DME.

**Table 2:** Demographics and clinical characteristics of DME and PDR cases and comparison to controls

	Controls	DME Cases	P	PDR Cases	P
Number	435	270		176	
Age (yrs)	66.5 ± 12.6	65.9 ± 10.6	0.491	62.7 ± 10.6	< 0.001
Duration (yrs)	12.6 ± 7.1	19.2 ± 8.8	< 0.001	19.1 ± 8.9	< 0.001
Female	48.5%	43.0%	0.151	35.2%	0.003
Hypertension	76.8%	88.1%	< 0.001	87.5%	0.003
Nephropathy	12.2%	24.4%	< 0.001	29.5%	< 0.001
HbA1c (%)	7.5 ± 1.5	8.8 ± 1.8	< 0.001	8.9 ± 1.9	< 0.001

For continuous variables, values are given as the mean ± standard deviation. For dichotomous variables, values are given as a %. HbA1c is a % of total haemoglobin. Duration = known duration of type 2 diabetes in years

**Fig. 1:**
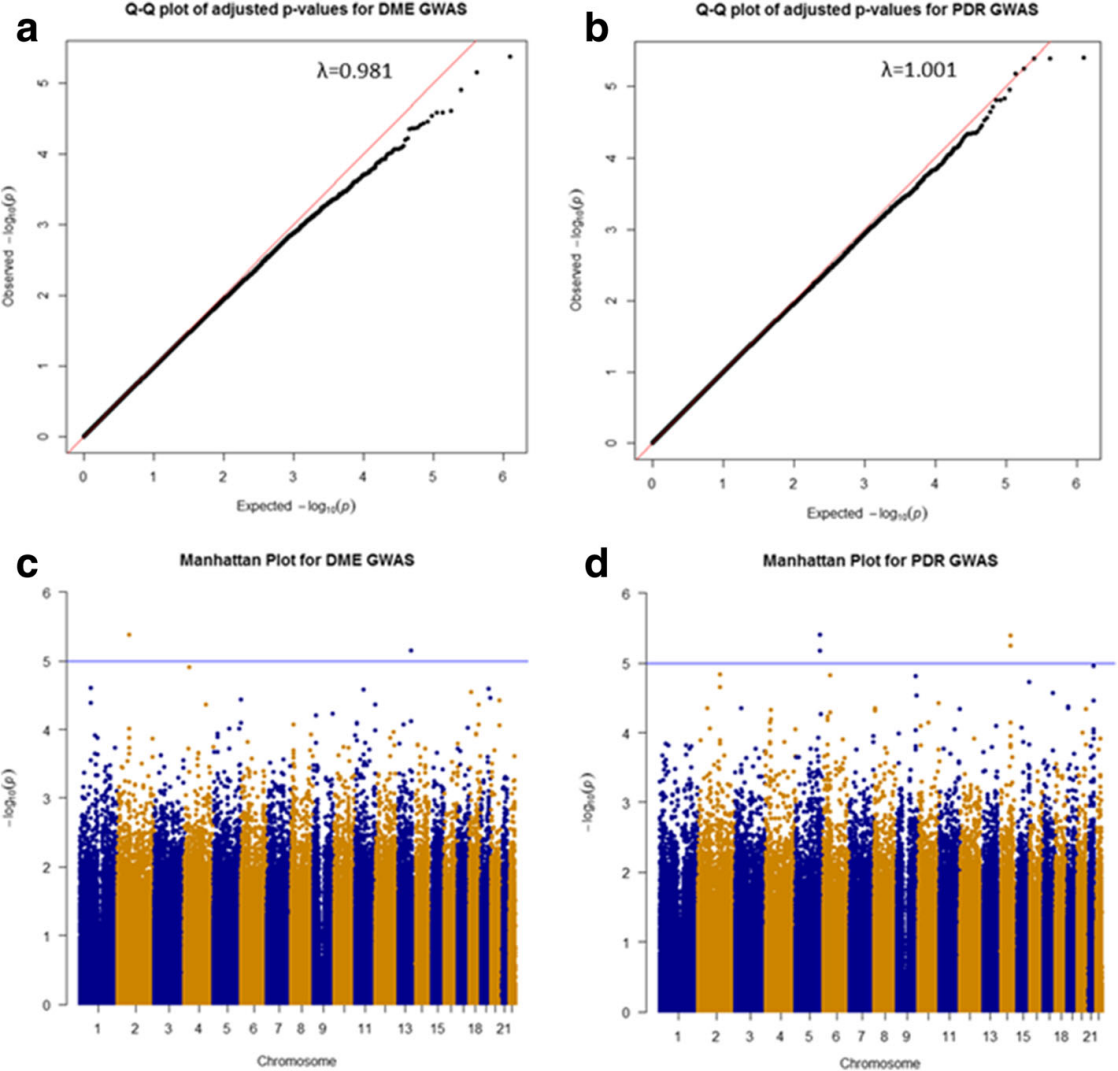
Q-Q plots and Manhattan plots for DME and PDR GWAS. Q-Q plots for (**a**) Diabetic Macular Edema and (**b**) Proliferative Diabetic Retinopathy GWAS. Manhattan plots for (**c**) Diabetic Macular Edema and (**d**) Proliferative Diabetic Retinopathy GWAS. The blue horizontal line represents suggestive association at *p* = 1.0 × 10^− 5^

The effects of population stratification were assessed through visualisation of the Q-Q plots (Fig. 1a and b) and lambda values (λ = 0.981, λ_1000_ = 0.943 for DME; λ = 1.001, λ_1000_ = 1.004 for PDR) and determined to be negligible following adjustment for the first three principal components. The Manhattan plots (Fig. 1c and d) show the association results for both DME and PDR analyses.

Association results for DME (Additional file 1) and PDR (Additional file 2) showed 7 variants reaching suggestive significance (Table 3). None of the SNPs reached genome-wide significance (*p* < 5 × 10^− 8^). In the DME analysis, only two SNPs displayed *p*-values < 1.0 × 10^− 5^. The highest ranked SNP was rs1990145 (*p* = 4.10 × 10^− 6^, OR = 2.02 95%CI [1.50, 2.72]) located in an intron of the *MRPL19* gene on chromosome 2. The second SNP, rs4771506 (*p* = 6.94 × 10^− 6^, OR = 1.97 [1.46, 2.64]), is on chromosome 13 near the *LINC00343* gene. In the PDR analysis the top ranked SNP, rs918519 (*p* = 3.87 × 10^− 6^, OR = 0.35 [0.22, 0.54]) and the fifth ranked SNP, rs918520 (*p* = 6.66 × 10^− 6^, OR = 0.34 [0.21, 0.54]) on chromosome 5, are near the long non-coding RNA gene *LOC285626* while the closest protein coding gene is *IL12B*. Three SNPs near the *NRXN3* gene on chromosome 14 are also suggestive of association with PDR.

**Table 3:** Top ranked SNPs associated with DME and PDR. SNPs with *p* < 1.0 × 10^− 5^ for each phenotype are shown

Phenotype	Chr	SNP	Position in hg 38 (bp)	Minor allele	MAF cases	MAF controls	OR (95% CI)	P	Nearest gene
DME	2	rs1990145	75,650,524	A	0.364	0.268	2.02 (1.50, 2.72)	4.10 × 10^− 6^	*MRPL19*
	13	rs4771506	105,843,651	C	0.325	0.263	1.97 (1.46, 2.64)	6.94 × 10^− 6^	*LINC00343*
PDR	5	rs918519	159,399,349	T	0.163	0.231	0.35 (0.22, 0.54)	3.87 × 10^− 6^	*LOC285626*
	14	rs1158314	79,961,389	G	0.515	0.400	2.16 (1.56, 3.00)	4.01 × 10^− 6^	*NRXN3*
	14	rs8004963	79,958,817	C	0.515	0.400	2.16 (1.56, 3.00)	4.01 × 10^− 6^	*NRXN3*
	14	rs11159428	79,950,890	T	0.512	0.399	2.13 (1.54, 2.95)	5.63 × 10^−6^	*NRXN3*
	5	rs918520	159,399,302	C	0.131	0.211	0.34 (0.21, 0.54)	6.66 × 10^− 6^	*LOC285626*

Odds ratios (OR) calculated with respect to the minor allele. *P* values are adjusted for age, duration of diabetes, sex, hypertension, nephropathy (defined as microalbuminuria or worse), HbA1c and the first 3 principal components. *Chr* chromosome, *MAF* minor allele frequency

We compared our results to the only other published GWAS of DR in Caucasians. SNPs associated with sight-threatening DR in the type 1 diabetes cohorts reported by Grassi et al. (2011) [14] were evaluated in the DME and PDR datasets. Although none of the SNPs reached formal statistical significance for the eight loci tested at *p* <  0.006, two SNPs showed a strong trend towards association with *p* ≤ 0.008 (Table 4). The chromosome 10 SNP rs12267418 near *MALRD1,* was associated with DME, but clearly not with PDR. Further, the chromosome 20 proxy SNP rs16999051 within *PCSK2* was associated with PDR (*p* = 0.007) and was also nominally associated with DME (*p* = 0.038). Both these associations are in the same direction as in the original report.

**Table 4:**
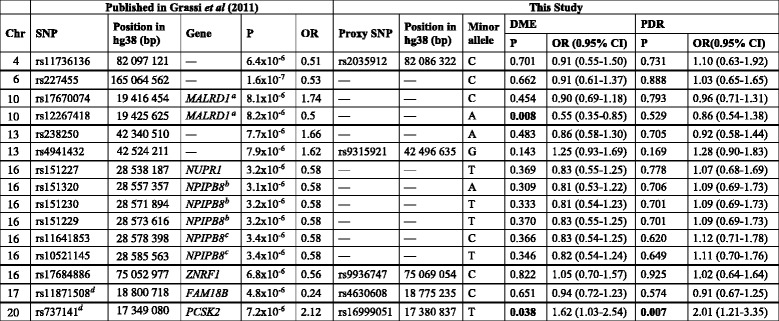
Comparison of GWAS findings for DME and PDR with a previously published GWAS for sight-threatening DR in a Caucasian population (Grassi et al., 2011 [14])

*p* < 0.006 was required for significance to account for the eight loci tested (as separated by horizontal bold lines). Nominally significant p-values (*p* < 0.05) are shown in bold. Proxy SNPs were used in this study when published SNPs were not available. Proxy SNPs were selected based on r^2^ value with the reported SNP in the CEU population of HapMap. 95% CI for the OR was not reported by Grassi et al., 2011 [14])

^a^In Grassi et al. 2011, the MALRD1 locus was labelled as *C10orf112* and *NPIPB8* was labelled as *CCDC101*^b^ and *SULT1A*^c^

^d^SNP with lowest *p*-value within 50 kb of the published SNP (no appropriate proxy SNP identified)

## Discussion

These GWAS analysed DME and PDR as separate phenotypes of the previously reported sight-threatening DR phenotype of Burdon et al. (2015) [13]. This is the first reported GWAS of DME in any ethnic group. Although no SNPs reached genome wide statistical significance, several suggestive loci have been identified for future investigation in other cohorts with DME phenotyping. The top ranked SNP for DME, rs1990145 is within the second intron of the mitochondrial ribosomal protein L19 (*MRPL19*). Although there have been no reports on this gene’s involvement with diabetic retinopathy, it is expressed in the retina (The Ocular Tissue Database [20]). Evidence suggests a key role for mitochondrial dysfunction in age-related macular degeneration [21]. Polymorphisms in this gene may result in mitochondrial dysfunction and associated eye pathology, with underlying subclinical phenotypes unmasked by conditions of stress such as diabetes and hyperglycaemia. At least three other MRP genes; *MRPL9*, *MRPL23*, and *MRPL39* map to genomic regions associated with retinitis pigmentosa [22] indicating the importance of this pathway in retinal pathology. The second ranked DME SNP, rs4771506, is approximately 80 kb from the long intergenic non-protein coding RNA 343 (*LINC00343*). The nearest protein coding gene is *DAOA*, D-amino acid oxidase activator, approximately 350 kb upstream from the SNP. It is difficult to hypothesise a functional role for this SNP or the nearest genes in DME and replication of these findings is clearly required.

This study is the first GWAS of PDR in Caucasians. We identified suggestive association of two SNPs within *LOC285626* and three SNPs within *NRXN3* with PDR. The presence of multiple SNPs at each locus with similar statistical evidence provides confidence in the findings, although, as for DME, replication and larger studies are clearly needed. *LOC285626* is approximately 35 kb upstream of the two chromosome 5 SNPs and encodes an uncharacterised, long non-coding RNA. The nearest protein coding gene is *IL12B* (Interleukin 12B), a further 33 kb upstream. *IL12B* is expressed in the retina (The Ocular Tissue Database [20]) and has been implicated in both type 1 [23, 24] and type 2 diabetes [25], although there are no prior reports of a role in diabetic eye disease. *NRXN3* (Neurexin 3) is located approximately 90 kb upstream from the three associated SNPs on chromosome 14 and is strongly expressed in the retina. The gene encodes multiple transcripts and although the exact function of each is unknown, neurexin proteins function as cell adhesion molecules and receptors in neurons. Genetic variants in *NRXN3* have been associated with increased waist circumference and obesity [26, 27], which are important risk factors for and features of type 2 diabetes [28, 29] and central obesity is a risk factor for DR [30, 31]. *NRXN3* polymorphisms are also associated with smoking behaviour [32], which in turn is a risk factor for age-related macular degeneration [33] although the contributory role of smoking to DR is less than for age-related macular degeneration. If *NRXN3* is confirmed to be important in PDR, there may be overlapping genetic risk factors for risk of type 2 diabetes and PDR.

None of the highest ranked SNPs in this study have previously been reported in other GWAS of DR [10–12, 14, 34]. All but one of these studies [14] were of non-Caucasian participants. If there is a genetic component to susceptibility which is linked with ethnicity, it could explain the difficulty in replicating results from these other studies. The lack of support for SNPs found in this current study with other published GWAS does not discount their possible association with DR. Replication analyses with larger numbers of participants would provide greater statistical power to confirm their association with PDR and/or DME. Similarly, molecular manipulation of cultured cell and animal models may be informative.

We have also directly compared the results of our DME and PDR GWAS to the only other DR GWAS of Caucasians [14]. Grassi et al. [14] reported a GWAS of severe DR in type 1 diabetes in Caucasian Americans, defined as PDR or DME. Similar to the current study, no variants were reported at the genome-wide significant level, however, several loci showed suggestive association (*p* < 1 × 10^− 5^). One of the top ranked variants, rs12267418, trended towards association with severe DR in the earlier report (*p* = 8.2 × 10^− 6^) [14] and with DME in the current study (*p* = 0.008, 8 loci tested). This SNP is located within an intron of the *MALRD1* gene on chromosome 10. Very little is known about the function of *MALRD1* (MAM and LDL receptor class A domain containing 1). Another recent study [35] on DR in a Chinese type 2 diabetic population included two SNPs within the *MALRD1* gene, although an association was not found (rs17670074 *p* = 0.27, rs9888035 *p* = 0.30). Further research is needed to understand the possible role of this gene in DR and whether ethnic differences or epigenetic effects might be responsible for the differing results.

We also show nominal association at the chromosome 20 locus near *PCSK2* reported by Grassi et al. Due to the use of different SNP arrays in the two studies, we report results for rs16999051 whereas the earlier report was for rs737141. The association of this SNP with PDR was very close to significance (*p* = 0.007) and the SNP also reached nominal significance with DME (*p* = 0.038). This may suggest some overlap in the genetic susceptibility to different diabetic ocular complications, but may also be due to the number of DME patients who also have PDR. This SNP is located within an intron of the *PCSK2* gene on chromosome 20. The proprotein convertase subtilisin/kexin-type 2 gene has been well-documented in its link to susceptibility to type 2 diabetes [36, 37].

This study compared type 2 diabetes participants with type 1 diabetic participants in Grassi et al. (2011) [14]. The similar trends at several SNPs may suggest that some loci are associated with DR regardless of diabetes type. Similarly, the DR associated SNP found in our original report [13], rs9896052, was found in the discovery type 2 diabetes cohort as well as the replication type 1 diabetes group, again suggesting that at least some DR risk factors are common between types of diabetes.

A strength of this study is the size of the original cohort relative to other DR studies [15], and the detailed clinical evaluation of the participants. Stratifying the sight-threatening phenotype of our earlier study [13] into DME and PDR provided more homogeneous groups for analysis. This study is comparable in size to other published GWAS for DR phenotypes and provides a useful breakdown of data from the previously reported composite phenotype of blinding retinopathy [13], although we have a limited ability to detect small effect sizes. A recognised limitation is that 93 participants have both PDR and DME, indicating that these phenotypes may not be independent even though we have conducted the analyses separately. This issue will affect any cohort of diabetic patients and can only be unravelled using larger cohorts and laboratory based functional testing. It is noteworthy that many participants have either DME or PDR, supporting the hypothesis that different factors lead to these different clinical phenotypes. Further value could be gained from the data by a meta-analysis with other reported studies, following harmonization of phenotypes. Imputation of un-genotyped SNPs may also reveal additional loci, however, most loci detected through imputation are also tagged by directly genotyped SNPs. Such analyses are likely to be of limited value in this small cohort. Replication cohorts are also required to fully evaluate the findings reported herein, particularly as genome-wide statistical significance was not reached. Relevant basic science studies are also merited.

## Conclusions

This is the first GWAS specifically targeting DME and PDR separately as ocular complications of sight-threatening DR. *MRPL19* and *NRXN3* have been identified as novel loci with suggestive association with DME and PDR respectively. Two long non-coding RNAs of unknown function (*LINC00343* and *LOC285626*) have also been highlighted as possible candidates for these common, blinding complications of diabetes, however, further genetic studies are warranted to confirm these findings. This study has also provided supportive evidence for previously reported loci, *PCKS2* and *MALRD1* in DR in Caucasians. Despite recent advances in treatment for diabetes related systemic risk factors and of local DME and PDR with anti-VEGF therapies and fenofibrate [38], many patients remain visually impaired. Studies such as this can lead to clearer understanding of important biological pathways relevant to disease and will be important tools in the global fight to prevent blindness from diabetes.

## Additional files

Additional file 1: Association results for Diabetic Macular Edema GWAS. Only SNPs with *p* <  0.05 are included in this file. (TXT 21762 kb)

Additional file 2: Association results for Proliferative Diabetic Retinopathy GWAS. Only SNPs with *p* <  0.05 are included in this file. (TXT 21943 kb)

## Abbreviations

Chr: Chromosome
DME: Diabetic macular edema
DR: Diabetic retinopathy
GWAS: Genome-wide association study
MAF: Minor allele frequency
N: Number of participants
NPDR: Non-proliferative diabetic retinopathy
OR (95% CI): Odds ratio (with 95% confidence intervals)
P: *P*-value, calculated probability
PDR: Proliferative diabetic retinopathy
SNPs: Single nucleotide polymorphisms
